# Operational adaptations of the trachoma pre-validation surveillance strategy employed in Ghana: a qualitative assessment of successes and challenges

**DOI:** 10.1186/s40249-019-0585-x

**Published:** 2019-08-27

**Authors:** Laura Senyonjo, Agatha Aboe, Robin Bailey, David Agyemang, Benjamin Marfo, Seth Wanye, Elena Schmidt, James Addy, Karl Blanchet

**Affiliations:** 1 Research team, Sightsavers, Haywards Heath, UK; 2 Clinical Research Department, London School of Hygiene & Tropical Medicine, London, UK; 3 Sightsavers Ghana office, Accra, Ghana; 4 Ghana Health Service, Accra, Ghana; 5 Department of Global health and Development, London School of Hygiene & Tropical Medicine, London, UK

**Keywords:** Trachoma, Pre-validation, Surveillance, Elimination, Case-finding, Surveillance strategy, Adaptation

## Abstract

**Background:**

In 2009 Ghana began to design a trachoma pre-validation surveillance plan, based on then-current WHO recommendations. The plan aimed to identify active trachoma resurgence and identify and manage trichiasis cases, through both active and passive surveillance approaches. This paper outlines and reviews the adaptations made by Ghana between 2011 and 2016. The assessment will provide a learning opportunity for a number of countries as they progress towards elimination status.

**Methods:**

A mixed methods approach was taken, comprising in-depth interviews and documents review. Between January and April 2016, 20 in-depth interviews were conducted with persons involved in the operationalisation of the trachoma surveillance system from across all levels of the health system. A three-tier thematic coding framework was developed using a primarily inductive approach but also allowed for a more iterative approach, which drew on aspects of grounded theory.

**Results:**

During the operationalisation of the Ghana surveillance plan there were a number of adaptations (as compared to the WHO recommendations), these included:

(i) Inclusion of surveillance of active trachoma in the passive surveillance approach, as compared to trichiasis alone. Issues with case identification, challenges in implementation coverage and a non-specific reporting structure hampered effectiveness;

(ii) Random selection and increase in number of sites selected for the active surveillance component. This likely lacked the spatiotemporal power to be able to identify recrudescence in a timely manner;

(iii) Targeted trichiasis door-to-door case searches, led by ophthalmic nurses. An effective methodology to identify trichiasis cases but resource intensive;

(iv) A buddy system between ophthalmic nurses to support technical skills in an elimination setting where it is difficult to attain diagnostic and surgical skills, due to a lack of cases. The strategy did not take into account the loss of proficiency within experienced personnel.

**Conclusions:**

Ghana developed a comprehensive surveillance system that exceeded the WHO recommendations but issues with sensitivity and specificity likely led to an inefficient use of resources. Improved targeted surveillance strategies for identification of recrudescence and trichiasis case searches, need to be evaluated. Strategies must address the contextual changes that arise because of transmission decline, such as loss of surgical skills.

**Electronic supplementary material:**

The online version of this article (10.1186/s40249-019-0585-x) contains supplementary material, which is available to authorized users.

## Multilingual abstracts

Please see Additional file 1 for translations of the abstract into the five official working languages of the United Nations.

## Background

Surveillance involves the continuing, systematic collection, analysis and interpretation of data for the planning, implementation and evaluation of public health practice [1]. An effective surveillance system can provide an early warning for potential public health emergencies or situations that require early intervention, help determine the impact of an intervention or track progress towards specific goals and assist in understanding the epidemiology of a disease, allowing priorities to be set and inform public health policies and strategies [1]. A system can include both active and passive components. Active surveillance requires specific action to obtain information as opposed to passive surveillance which involves the reporting of information that is being collected through the existing health system [2].

Trachoma, one of the neglected tropical diseases, is a chronic, progressive disease that if left untreated can lead to blindness. The global goal for trachoma is the elimination as a public health problem by 2020 [3]. The World Health Organisation (WHO) uses the simplified grading system, denoting five signs of the disease [4], focusing on two grades to monitor trachoma elimination efforts. These are trachomatous inflammation—follicular (TF), defined by five or more follicles, each at least 0.5 mm in diameter, in the central part of the upper tarsal conjunctiva (a sign associated with ocular *Chlamydia trachomatis* infection); and trachomatous trichiasis (TT), defined as at least one eyelash touching the eyeball or evidence of recent removal of in-turned eyelashes (a sign associated with risk of current, progressive loss of vision) [5]. In order to achieve elimination, countries need to provide evidence that all evaluation units (population of between 100 000–250 000), often districts, have achieved and maintained a TF prevalence of less than 5% in children aged 1–9 years and a TT prevalence (unknown to the health system) of less than 0.2% in adults aged 15 or over. Countries must also have an adequately resourced surveillance system in place that is able to identify and manage incident TT cases [6].

Trachoma surveillance has been defined by WHO as the “monitoring and evaluation activities that assess the outcome of a trachoma elimination programme, conducted after elimination prevalence targets appear to have been achieved, in a defined trachoma endemic area” [7]. Continued surveillance is important as elimination of trachoma as a public health problem does not necessarily equate to interruption of transmission of *C. trachomatis*, the causative agent of trachoma [8] and therefore in an elimination setting there is an unquantified risk of recrudescence. Trachoma surveillance can be broken down into two stages, activities up to the preparation of the dossier for validation of elimination of trachoma, termed “pre-validation surveillance” and activities occurring after validation, termed “post-validation surveillance”. Pre-validation surveillance aims to ensure that elimination thresholds are sustainably achieved, detect any possible re-emergence of disease (recrudescence) in a timely manner and continue to identify incident (and recurrent) TT cases [7]. Guidance to date has primarily focused on pre-validation surveillance.

### The WHO guidance for pre-validation surveillance

Initial WHO guidance around the optimal method for pre-validation surveillance for trachoma was published in 2008. It followed the outcomes of the technical consultation on trachoma surveillance [2], later revised following the 2014 consultation [7]. In 2008, the report outlined minimum requirements and guidelines for implementation of a three-year surveillance period once elimination thresholds had been achieved. This consisted of monitoring TF prevalence in school entrance-aged children, or all children aged 1–9 years depending on school enrolment figures and TT in adults aged 40 and above in select sites [2]. Specifically the key recommendations outlined in 2008 included:

#### Passive surveillance

- Countries were to have an adequate system in place to be able to collect and analyse the number of TT cases identified and operated on or managed per year. This should have included reporting on the number of individuals that have refused surgery and recurrent cases.

#### Active surveillance

- Trachoma surveillance for TF was to be conducted in two selected sites (population of 1000–2000) per endemic district per year biased to those suspected to be trachoma endemic and the least developed. The selected site was either to be a school or community, with different sites to be selected each year. All school entrance-aged children should have been examined for TF where school attendance is > 90% and there is no gender bias. Where this is not the case, a minimum of 50 children in the community (5–6 ± 2 years of age) should have been assessed, or if feasible all children in the community should have been examined. If TF was greater than 5% in the examined children, then examination should have been extended to all children in the community (aged 1–9 years), then if indicated, the sub-district and ultimately neighbouring sub-districts.
- In the same two selected sites per endemic district per year as for the TF assessment, all individuals aged 40 or older were to be assessed for TT [﻿2﻿].

Following the deliberations in the 2014 technical meeting on trachoma surveillance, the guidance changed, moving away from recommending active on-going surveillance for TF through monitoring of selected sites and instead the implementation of a population-based survey at the level of the district (population 100 000–250 000). It also re-emphasised the need to ensure an appropriately resourced surveillance system is in place to be able to identify and manage incident TT cases [﻿7].

### Trachoma elimination in Ghana

Trachoma was endemic across the Northern and Upper West regions of Ghana as evidenced by a number of population-based prevalence surveys conducted between 2000 and 2003. The results highlighted a number of districts that had trachoma of public health significance, including four with a TF and trachomatous inflammation—intense (TI) prevalence of over 10% in children aged 1–5 years [9]. As a result the Ghana Health Service (GHS) implemented a successful trachoma control strategy, which involved disease mapping and implementation of the full SAFE strategy (surgery, antibiotic distribution, facial cleanliness and environmental improvements). By 2009, all districts across the trachoma endemic Northern and Upper West regions had reached the elimination thresholds (or for TT, working to reduce final backlog) and all districts were able to transition from intervention to surveillance activities [10, 11]. In 2009–2010, with support from WHO and other partners, GHS developed a pre-validation surveillance plan, based on WHO recommendations at the time and in line with the integrated disease surveillance and response (IDSR) framework [12]. This was piloted in 2011 before scale up and roll out between 2012 and 2014 [9]. In 2015 and 2016, GHS conducted a population-based survey at the level of the district to provide the evidence that elimination of trachoma as a public health problem had been achieved [10].

As outlined in the GHS surveillance plan for trachoma, published in 2010 [13], the main objective of the surveillance was to provide evidence for the elimination of trachoma. Specific objectives were to be able to detect and manage TT cases, integrate TT surveillance into the IDSR system and to detect and respond to any potential resurgence of active trachoma (TF), alongside the collection of key water and sanitation indicators [13].

The WHO recommendations and guidance were primarily based on expert opinion and expectations of a suitable approach. Ghana was one of the first sub-Saharan African countries to implement a pre-validation surveillance plan and therefore the approach had to be adapted to fit local context, systems and understanding. Reviewing the Ghana experience is useful in helping to understand their experience in implementing a trachoma surveillance system and adaptations they made but also what is practical and effective in an elimination context. This learning is particularly useful as a number of countries now progress towards elimination status. This paper outlines and reviews the adaptations made to the pre-validation surveillance system employed by Ghana between 2011 and 2016.

## Methods

### Data collection

**Fig. 1:**
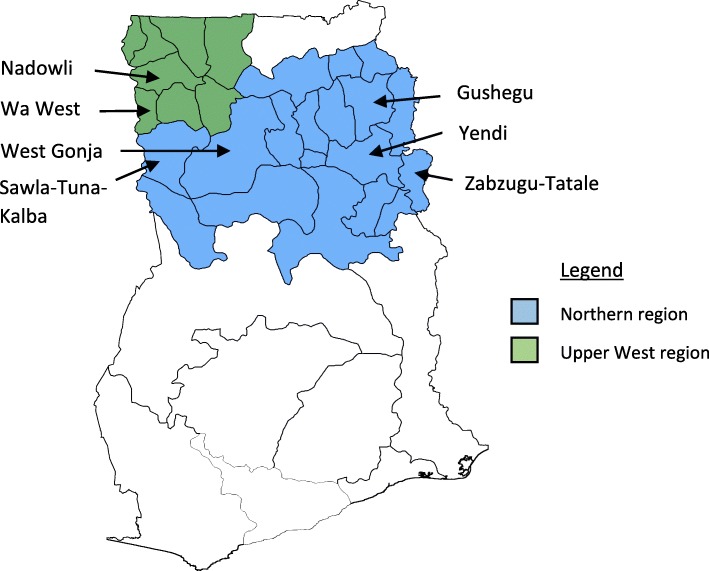
Map of the study area, Northern and Upper West regions of Ghana. Districts indicated are referenced in the paper; adapted from an open source map retrieved from www.mapmaker.com

A mixed methods approach was undertaken, comprising in-depth interviews and documents review. This approach was appropriate in order to understand users’ experiences and perspectives narrated through qualitative interviews. This information was verified through a review of key documentation outlining the activities and results of the trachoma surveillance approach in the Northern and Upper West regions of Ghana, Fig. 1. Formal observations were not included as part of the evaluation as activities which are part of the pre-validation surveillance stage had largely ceased by the time of data collection.

Between January and April 2016, a total of 20 semi-structured interviews were conducted with persons involved in the operationalisation of the trachoma surveillance system, across all levels of the health system from the national level to general health workers, Table 1. The cadre of staff chosen were able to provide a comprehensive view as to the successes and challenges in delivering the trachoma surveillance strategy in Ghana. These persons had been instrumental in the design and management of the surveillance system or are key actors involved in the successful implementation of the activities, either as ophthalmic specialists or generalists working within the health system. District disease control officers (DCO) and ophthalmic nurses (ONs) were chosen to represent geographically dispersed districts across the two regions and as active or more experienced personnel in post during the study period (2011 to 2015).

**Table 1:** Summary of persons interviewed

Level	Number
National focal persons responsible for trachoma and general surveillance activities in Ghana	2
Regional focal persons responsible for trachoma and general disease control in Upper West and Northern regions	4
District disease control officers chosen to represent districts across the two regions and as active DCOs during the time period of interest (2011 to 2015)	4
Ophthalmic nurses were chosen amongst the most experienced in office and a representation of districts across the two regions	4
General health workers	6

Interview guides were developed using the 2001 Centers for Disease Control structured checklist for the review of public health surveillance systems [14]. The interviews covered information on the description of the trachoma surveillance system, resources required and operational gaps, assessment of adequacy and usefulness of the surveillance system, quality assessment of system performance and finally information on the integration and co-ordination between the trachoma surveillance system and other surveillance structures or aspects of the health system. All Interviews of the health workers were conducted in English. Interviews were audio-recorded and transcribed verbatim.

### Data analysis

A three-tier thematic coding framework was developed using a primarily inductive approach utilising the interview guide to structure the initial coding. The methodology also allowed for a more iterative approach, which drew on aspects of grounded theory, allowing for new themes and ideas to develop from the interviews. The final themes of the analysis emerged around the adequacy and usefulness of key adaptations and the successes and challenges in implementing them in the Ghanaian context.

Coding was facilitated using NVivo qualitative data analysis software (Version 10, QSR International Pty Ltd.; www.qsrinternational.com/nvivo). The findings and conclusions were validated in collaboration with key stakeholders from GHS and partners in Ghana.

The document review included international and national trachoma surveillance plans and guidelines [13], WHO trachoma expert surveillance meeting reports [2, 7] and the Ghana trachoma elimination dossier [9]. The documents were reviewed in conjunction with the findings from the in-depth interviews to provide context and an understanding of how and why relevant operational adaptations to the surveillance strategy were conducted.

Written informed consent was received from each participant interviewed. Anonymised illustrative quotes were included as appropriate in the manuscript. Ethical approvals for the study were obtained from the GHS ethical review committee (03/07/15) and the London School of Hygiene and Tropical Medicine Review Board (reference: 10285).

## Results

GHS designed the methodology for their trachoma pre-validation surveillance system based on the recommendations of the WHO guidelines set out in 2008 [2] and the relevant goals of the existing IDSR strategy [12]. During the operationalisation of the Ghana surveillance plan there were a number of adaptations, in part to address the local context, financial and logistical constraints but also as a result of the interpretation of the scientific rationale for activity implementation. The adaptations put in place, the rationale for doing so and the effectiveness of the approach are explained below.

1. Inclusion of surveillance of active trachoma (TF) in the passive surveillance approach

A key objective of the trachoma surveillance system is to identify resurgence of infection, or TF as a proxy. The WHO recommends the use of the passive surveillance system for TT only [2]. However, the GHS designed a more comprehensive passive surveillance system, which included a network of community volunteers screening for both the active and chronic stages of trachoma. All health workers were to have an index of suspicion for trachoma, at all levels of the health system and irrespective of the condition the individual was attending for. Trained ONs were in place to detect and treat trachoma cases presenting at the eye care clinic. The ON also had the responsibility to confirm and if appropriate, clinically manage all suspected cases of trachoma reported by the community volunteers or health workers [9].

The case definition for suspected trachoma was defined as an individual with ‘red sticky eyes who also complains of eye pain or itchiness’, a proxy for active trachoma or ‘eye lash touching the eye’ to identify a case of trichiasis (TT). The non-specific case definition for active trachoma was used, as opposed to the WHO TF definition, as it was designed to put emphasis on simplicity, for ease of use for non-ophthalmic specialists (as everting the eyelid and identifying follicles is relatively difficult to do and requires training) and sensitivity, to ensure as many cases as possible were identified.

“*You just give them a loose definition that they can use, like if you say anybody with eye problems that is a loose definition, so that they can push all the cases, all the eye problems and we will now be able to use the real case definition to see whether it is trachoma or it’s not trachoma.*” (District disease control officer)

However, there were a number of challenges impeding the effective passive surveillance of active trachoma, including issues with case identification, de-prioritisation of trachoma as a health problem, challenges in implementation coverage and a non-specific reporting structure. Firstly, although the case definition was designed to be sensitive, not all infections with *C. trachomatis* are symptomatic or result in what can be perceived as a reddening of the eye. Therefore, the case definition will still miss cases in the community. Secondly, a red eye can be caused by common conditions such as non-chlamydial conjunctivitis [15].

“*They are eye problems, they don’t refer [just] trachoma cases. They refer anything that is [an eye problem], if I come to you and say [I} am having problems with my eye they refer, they don’t know trachoma*.” (Ophthalmic nurse)

However, although the risk of the approach was an over-diagnosis of suspected trachoma and pressure on the surveillance system to verify and manage cases, in reality few cases were actually reported using this system.

“*Because since after the training none of them, not even a single one in this district has referred a TF case to me, not even one. I don’t want to believe that they have not had even one, I want to believe maybe they are not able to identify it*.” (Ophthalmic nurse)

Further reasons reported for the ineffectiveness of the system were due to a deprioritisation of the condition at the community and health facility level, coupled with constraints in the roll out of the system across all health facilities and communities.

“*If the person comes and complain of an eye disease then the person will be screened for trachoma, but if they come for any other thing we normally don’t do*.” (General health worker)

Financial and logistical constraints effectively hampered the coverage of the surveillance implementation. Although a total of 30 health workers per district and one health volunteer per village were trained in trachoma surveillance [9], the numbers were insufficient to ensure adequate geographical coverage and the successful implementation of the passive surveillance approach.

“*There was no need [in] training just a few health workers and then we expect that the whole district will be actively involved, so it’s a good protocol except that it involves money and every health worker needed to be at least trained or to be oriented in trachoma for the passive surveillance to succeed*.” (National level informant)

Further, there were issues in the utility of the trachoma data generated using the passive surveillance approach. GHS ensured that trachoma was included in both the community-based surveillance (CBS) reporting form (retrospectively added to forms using a stamp of an eye to capture trachoma cases) and the IDSR. The CBS form is a simple form for a multitude of diseases of interest, which the community volunteers used to report to the health facility, on a monthly basis. There are two IDSR forms, a weekly form for epidemic prone diseases and diseases of interest and a monthly form for the surveillance of other specific disease of interest including trachoma [12]. The IDSR form allows for the compilation of patient data at the level of the health facility, consolidated from the sub-district to district level, where it is inputted into the District Health Management Information System 2 (DHMIS2), an electronic platform for data management. However, despite the adaptations of the forms, none differentiated between the various signs of trachoma. A person with ‘red eye’ would be recorded the same as someone with ‘eyelashes touching the eyeball’. Further reporting challenges included a lack of differentiation in the reporting system between suspected or confirmed cases of trachoma and incomplete reporting, particularly as active trachoma surveillance activities were not routinely reported in the DHMIS2. Therefore, although the GHS made efforts to adapt the reporting structure to incorporate trachoma, this was not adequate to be able to use and interpret the data collected in order to illicit an appropriate action-response.

“*The DHMIS is being used a lot but when you cross check the figures there they don’t really reflect what [is going on].*” (Regional level informant)

In general, the consensus among study participants was that the passive surveillance approach was more appropriate for trichiasis or TT surveillance as opposed to active trachoma or TF. TT has a more specific case definition and it is easier to diagnose without specialist expertise, has a more specific case definition and those with trichiasis have a greater incentive to seek care, as it is painful and can lead to blindness if not managed appropriately.

2. Random selection and increase in number of sites selected for active surveillance component

In order to identify TF resurgence in a timely manner, WHO, at the time, recommended the use of active surveillance. The guidance recommended monitoring school-aged children in two sites per district, purposefully selected biased to areas that are the most likely to be trachoma-endemic or with known risk factors for trachoma [2]. The assumption being that there is potential to use risk factors or knowledge of an area to purposefully select communities that would be markers for potential resurgence of *C. trachomatis* infection [6]. In contrast, the approach adopted by GHS involved randomly selecting the communities to ensure a representative sample selected with no bias, more similar to a survey approach.

*“We have to randomly select them because it is a study, so you cannot just hand pick [communities] because probably I am from here so I can pick this my favourite hometown or something.”* (Regional level informant)

In two randomly selected communities (villages) per year, GHS conducted a door-to-door case search and examined children aged 1–9 years for TF and adults aged 15 and above for TT. Additional screening of children aged 1–9 years in five schools (pre-school and primary school) and the village where the school was sited, were also conducted in each district. All sites were sampled without replacement. The number of sites visited was higher than the two sites (schools or community) per district recommended by the WHO. To ensure higher coverage, initial efforts were made to monitor seven sites per sub-district but the related financial and time costs meant this was unsustainable.

A total of eight sites with a TF prevalence of greater than 5% in children aged 1–9 years, were identified during 2012 and 2014, two in Zabzugu-Tatale, one in Gushegu, two in Sawla-Tuna-Kalba, one in West Gonja, one in Wa West and one in Nadowli. Children in the neighbouring villages (up to four), identified by the health facility worker, were subsequently examined for signs of TF. The plan was for the search to be extended to the sub-district, as per WHO recommendations, if the neighbouring communities also had a TF prevalence of ≥5% in children. This was a compromise, up to four villages would be able to provide an indication of any wider transmission or potential resurgence but limit use of resources, time and costs. All of the neighbouring communities examined had fewer than 5% of children with TF. Three rounds of mass antibiotic distribution were given to each of the eight communities.

3. TT door-to-door case search, led by ONs, targeted to an evaluation unit that had failed to meet the TT elimination thresholds.

The WHO recommendations mention the use of door-to-door TT case searches as a useful approach to identify TT cases, either as a standalone or an integrated activity with other ophthalmic campaigns. However, there is little clarity on who should do it or where it should be conducted. In Ghana it was used as a supplemental activity and involved the systematic door-to-door, community-by-community case search for TT cases led by the ONs. Door-to-door case searches had been conducted as routine as part of the surgical component of the SAFE strategy, sometimes integrated with other case searches for example guinea worm or cataracts and the practice continued during the surveillance stage. However, during the surveillance period, Ghana was able to better target their resources for trachoma case searches to districts that had failed to reach the TT elimination thresholds during the impact assessments or the pre-validation surveys. The strategy was determined to be a necessary approach to quickly identify and manage TT backlogs.

Intensive counselling of patients found to have TT, with an offer of immediate surgery helped ensure improvement in surgical uptake [9]. Although very useful in rapidly finding a large number of TT cases, the strategy was resource intensive, expensive and time consuming and this impacted on the sustainability of the approach, ultimately affecting coverage.

“*It’s very costly …*. [*it] is the only way you can get the TT cases, but then it’s very costly doing the house-to-house case search*.” (National level informant)

Some of the most intensive efforts for TT door-to-door case searches was in Yendi district, in the Northern region, which failed to reach the TT elimination threshold following the pre-validation survey in 2016 [10]. In Yendi, a total of 72.1% of the expected district adult population (over 15 years old) was examined [9].

“*It is very, very labour intensive number one, you needed a lot of money for this house to house, community-by-community case search and it got to a time we said enough was enough.”* (National level informant)

In Yendi, the effectiveness of the TT door-to-door case search in the endgame is corroborated by the comparative analysis of the TT data reported through different strategies, namely the routine passive surveillance approach, the pre-validation surveys and the TT door-to-door case search. During 2011 and 2015, a total of 33 trachoma cases were identified through the passive surveillance system, across the previously trachoma endemic Northern region [9]. Following the pre-validation surveillance survey conducted in 2015–2016, Yendi was determined to have a TT prevalence unknown to the health system of 0.36% (95% CI: 0.0–1.01) in adults aged 15 and above, relating to a backlog of 417 people [10]. A total of 90 TT cases (19 males and 71 females) were subsequently identified through the thorough door-to-door case search and examination of a total of 61 225 individuals (0.15%). The age of TT patients identified ranged upwards from 28 years, with only a few cases found in individuals below the age of 50 years [9]. The passive surveillance system used in Ghana failed to adequately identify TT cases, whilst the survey approach indicated a higher TT backlog than was identified using the door-to-door case search. The Ghana experience highlights the benefits of conducting a comprehensive case search for a rare event such as TT in an elimination setting. Not only does it enable the determination of reliable TT estimates but has the added advantage of being able to immediately provide services for the timely management of cases identified.

4. Buddy system to support technical skills in an elimination setting

The WHO guidelines are activity orientated and provide little operational guidance in regards to some of the key deliberations countries may need to consider in the endgame, such as maintaining technical skills in a changing epidemiological environment, with ever decreasing case numbers.

The functionality of the Ghana trachoma surveillance system heavily relied on the technical expertise of the ON. The GHS policy is for an ON to be stationed at each eye care clinic in every district hospital. In an elimination setting, newly trained ONs were deployed into an environment with few trachoma cases and therefore have difficulty in gaining and maintaining skills, especially in TF diagnosis and TT surgeries. Based on an experience of misclassification of a community due to incorrect diagnosis of TF by an inexperienced ON, GHS introduced a buddy system, whereby an experienced ON was paired up with an inexperienced ON in order to support and provide mentorship. In some districts, a similar system was also set up in regards to trichiasis surgeries with an experienced surgeon supporting ONs responsible for surgeries in their district.

“*If they are working together then the person will be learning, but if the fellow is new and has been given a district to man alone, then they have difficulty identifying TF in particular*.” (Ophthalmic nurse)

“*Of late trachoma is not something that is key or obviously seen now. The new officers that are coming now if you don’t orientate [them] that means even case detection will be an issue because automatically they will not be able to know what it is to look out for*.” (District disease control officer)

The buddy system provided assurances to GHS of the quality and validity of the diagnoses. However, consideration must be given as to the potential for the experienced ONs to lose their proficiency in diagnosing TF or even in conducting surgeries. During the certification process of trachoma graders (ONs) for the pre-validation surveillance surveys [10], a number of the more experienced ONs failed to reach the certification criteria and therefore it seems they had lost the diagnostic skills acquired when trachoma was more prevalent in the communities they served. A potentially more pertinent concern over deskilling is in relation to TT surgeries, in an environment with an ever-decreasing number of TT cases. Currently ONs are responsible for performing trichiasis surgeries in their districts, in many cases only one or even no surgeries a year. Although there is no formal guidance on the number of surgeries required per year to maintain TT surgical skills, the expectation is that the number of surgeries being performed by an ON in each district would be insufficient to be able to adequately retain skills [16]. No formal audit of surgical skills was conducted during this time period [9].

A summary of the analysis is presented in Table 2.

**Table 2:** Summary of the adaptations to the Ghana trachoma pre-validation surveillance system

No	Challenges in surveillance	Recommendations from WHO (2008 guidance)	Adaptations introduced by Ghana Health Service
1	How to identify resurgence of active trachoma (TF) using the passive surveillance system	None, concentrate on identification of TF through active surveillance approaches	Monitoring of active infection is integrated as part of the passive surveillance approach; a non-specific case definition was used initially but all suspected cases were expected to be verified by ophthalmic nurses
2	Site selection for active surveillance that would ensure resurgence of active trachoma identified in a timely manner	Purposeful selection of two sites per district for monitoring of TF in school-aged children, biased to sites at the most risk of trachoma	Screening of children aged 1–9 years in two randomly selected communities and five schools per district per year
3	Identify and manage TT backlog, especially where failure to do so may threaten TT elimination thresholds	Countries to have an adequate system in place to be able to collect and analyse the number of TT cases identified and managed (operated on) per year. Methodologies for collecting these data include door-to-door TT case search either as a stand-alone activity or co-ordinated with other ophthalmic campaigns	Intensified TT door-to-door case search, led by ONs. This was targeted to specified districts that had failed to meet the TT elimination thresholds as determined through impact assessments or pre-validation surveillance surveys
4	Deskilling of staff and new staff entering a setting with few cases to acquire skills	None	Buddy system to support technical skills

## Discussion

Ghana implemented their trachoma pre-validation surveillance strategy between 2011 and 2016, being one of the first countries in Africa to develop and implement such a surveillance plan. In general, the strategy closely followed the 2008 WHO recommendations, employing both passive and active surveillance approaches, with a population-based survey providing the ultimate evidence that TF and TT elimination thresholds had been maintained and validation of elimination of trachoma as a public health problem achieved. However, GHS employed a number of adaptations to the WHO surveillance recommendations at the time, with varying success.

Issues with the sensitivity and specificity of the passive surveillance system impeded the achievement of the surveillance objectives and resulted in an inefficient use of resources. In particular, monitoring of TF through the passive surveillance system was inadequate and compromised the interpretation and utility of the TT data. There were four key limitations, insufficient numbers of health workers and community volunteers trained on trachoma resulting in low coverage of the surveillance activities, deprioritisation of trachoma as a health problem, the non-specific case definition for active trachoma and insufficient differentiation in the reporting of suspected and confirmed cases and active and chronic signs. The passive surveillance system should be simplified and focus on strengthening the identification of unknown or incident TT cases.

The monitoring of a limited number of sites per year to identify resurgence of active trachoma is unlikely to have the power to systematically detect recrudescence in a timely manner. There were too few communities assessed per year to provide the power for a survey approach and the chances of randomly selecting any areas of possible resurgence is low. However, the purposeful selection of communities as recommended by WHO at that time, with the expectation they would be markers for potential resurgence of *C. trachomatis* infection is also very difficult, as trachoma community risk factors are not well understood and there are limited data on known risk factors that are available for every community. With such small sample sizes as used in Ghana, this could result in data unduly influenced by chance effect [7]. As a result, WHO no longer recommends the active surveillance of limited sites to monitor resurgence. Instead the focus is on the implementation of a population-based survey approach (approximately 30 clusters), to determine if TF (and TT) elimination thresholds have been maintained since MDA was stopped [6]. This approach has more power to be able to detect any resurgence of active trachoma at the level of the evaluation unit but still likely lacks the spatiotemporal power to be able to detect with any certainty, resurgence at any lower resolution. However, there is potential to utilise serology and PCR-based markers for trachoma to help identify communities at possible increased risk of recrudescence, possibly through the integration of these indicators into population-based surveys [8]. The targeted surveillance of communities identified with persistent *C. trachomatis* infection and or high seropositivity in children, could be a strategy for post-elimination surveillance.

A door-to-door TT case search, also used elsewhere to great effect including Morocco [2], is an effective approach to identify TT cases but is expensive (as viewed by GHS) and time-consuming, especially when using specialist technicians as in Ghana. With limited resources, even with external donor support, it is important to focus this strategy on communities or areas where the suspected burden of remaining TT cases reside. However, there is currently difficulty in identifying priority areas. Ghana utilised the findings of the impact and pre-validation surveillance surveys to determine where an exhaustive case search was required. However, TT is a rare event in a peri-elimination setting and an inadequate precision of survey estimates results in relatively large stochastic variations to TT prevalence estimates, resulting in prevalence estimates with wide confidence intervals [17]. To overcome this one would require a large sample size to be able to determine prevalence estimates with the necessary degree of precision, such as a TT only survey [18]. As evidenced in Yendi, there was a large discrepancy between the TT backlog estimate calculated through the data derived from the population-based survey compared with the data from the door-to-door case searches. Further, even if a district or evaluation unit does achieve TT elimination thresholds as evidenced by a survey approach, there is likely heterogeneity in community-level prevalences that get hidden by the evaluation unit level estimates. With the weaknesses in utilising a survey approach and the suggestion it may still miss significant TT backlogs, it may be that countries need to use alternative approaches to determine or prioritise areas for TT case searches. There is currently little evidence as to trends in geospatial distributions of TT cases in a peri-elimination setting but it has been postulated that the remaining TT cases are most likely in the more remote communities furthest from the static eye care services. Further research would be useful to test this hypothesis.

A cheaper alternative option may be to further strengthen the community volunteer surveillance network to identify TT cases and simultaneously determine if TT elimination thresholds have been achieved, as shown in Kenya [19], Tanzania [20] and elsewhere. There is merit in suggesting that Ghana would have been better placed to have strengthened the passive surveillance system by focusing on TT only and potentially better incentivise the community volunteers to identify and refer TT cases. Globally, the guinea worm programme has utilised a comprehensive network of community volunteers and a high degree of public awareness in order to be able to identify a suspected case of guinea worm and have structures in place for immediate reporting and follow-up of cases and contacts [21]. Volunteers were given monetary incentives to report suspected cases of guinea worm. Although not using monetary incentives for finding TT cases, Tanzania trialled a training of community volunteers to use standardised screening questions and cards to identify TT cases during mass drug administration campaigns. Although successful in improving the number of cases identified as compared to the standard approach, there were a significant number of false positive cases identified [20]. Using monetary incentives is only likely to exacerbate issues with specificity and a balance within the system would be required to ensure cases referred are true TT cases.

Strategies to maintain technical skills of those involved in diagnosis of trachoma and TT surgeries is an important consideration in the endgame. Ghana implemented a buddy system using more experienced ONs to support and mentor less experienced persons. However, there was an assumption that all ONs maintained skills even though they too were seeing fewer cases, an assumption challenged during the training of graders for the pre-validation surveillance surveys. Loss in proficiency of surgical skills is also a key issue and it is important any surveillance system facilitates the maintenance of highly skilled surgeons. Rather than having an ON in each district with the responsibility to conduct limited numbers of trichaisis surgeries in their administrative area, it is perhaps optimal to keep the surgical skills within a few surgeons. They could be stationed in the region as a roving team that conduct all surgeries, thereby maintaining skills. If there are inadequate numbers of surgeries per year, additional training could also be conducted using the human eyelid analogue device for surgical training and skills reinforcement for trichiasis (HEAD START) [22].

## Conclusions

Countries have utilised a variety of approaches for pre-validation surveillance of trachoma, from door-to-door or school-based case searches, population based or school-based surveys, sentinel sites, health facility-based registers or case notification and border screening [2, 23]. However, in the elimination endgame, when resources are ever limited, it is important countries focus their surveillance activities on the most effective approaches that provide the evidence to achieve the surveillance objectives. Ghana developed a comprehensive surveillance system that exceeded the WHO recommendations but issues with sensitivity and specificity, especially for the monitoring of active trachoma resurgence, likely led to an inefficient use of resources. Some of the issues highlighted through the experience in Ghana have since been addressed in the 2014 revision of the WHO guidance on trachoma surveillance. Countries are currently offered support to conduct cross-sectional prevalence-based surveys using appropriate quality-assured and quality-controlled systems. However, improved targeted surveillance strategies, including use of serology and infection data to help identify communities at risk of recrudescence and methods to identify priority areas for TT door-to-door case searches, need to be evaluated. Further consideration also needs to be given during transition planning (from intervention to elimination) as to the adaptations to the surveillance strategy required to address the contextual changes that arise as a result of transmission decline, such as a loss of surgical skills.

## Additional file

Additional file 1: Multilingual abstracts in the five official working languages of the United Nations. (PDF 267 kb)

## Data Availability

The datasets used and/or analysed during the current study are available from the corresponding author on reasonable request.

## Abbreviations

CBS: Community based surveillance
DCO: Disease control officer
GHS: Ghana Health Service
HEAD START: Human eyelid analog device for surgical training and skills reinforcement for trichiasis
IDSR: Integrated disease surveillance and response
ON: Ophthalmic nurse
TF: Trachomatous Inflammation—follicular
TT: Trichiasis trachomatous
WHO: World Health Organisation
