# Supplementary material for: Operational adaptations of the trachoma pre-validation surveillance strategy employed in Ghana: a qualitative assessment of successes and challenges
Source: Infect Dis Poverty. 2019 Aug 27;8:78. doi: 10.1186/s40249-019-0585-x (PMC6712645; doi:10.1186/s40249-019-0585-x)
Supplement: Supplementary file 1 — Multilingual abstracts in the five official working languages of the United Nations. (PDF 267 kb) [file 40249_2019_585_MOESM1_ESM.pdf]

Translation of the abstract into the five official working languages of the United Nations

التعديلات التشغيلية لاستراتيجية مراقبة ما قبل التحقق من التراخوما المستخدمة في غانا: تقييم نوعي للنجاحات والتحديات

لورا سِنيُوهو، آجثا أبو، رابن بالي، ديفد آجْيِمانْج، بنيامين مارفو، سَث وايْن، ألَنا اشمِدت، جايْمز آدي، كارل بِلانشِت

ملخص
الخلفية: _ في عام 2009 ، بدأت غانا في تصميم خطة مراقبة ما قبل التحقق من التراخوما ، بناءً على توصيات منظمة الصحة العالمية الحالية آنذاك. تهدف الخطة إلى تحديد انبعاث التراخوما النشط وتحديد وإدارة حالات الإصابة بمرض النمو الداخلي لرموش العين (نمو الرموش داخل الجفن)، من خلال مقاربات المراقبة الفعالة، وغير الفعالة. تحدد هذه الورقة، وتستعرض التعديلات التي أجرتها غانا بين عامي 2011 و 2016. سيوفر التقييم فرصة تعليمية لعدد من البلدان أثناء تقدمهم باتجاه القضاء على المرض.

أساليب: تم إتباع نهج مختلط ، حيث تضمنت مقابلات عميقة، ومراجعة وثائق. بين كانون الثاني ونيسان ٢٠١٦، أجريت 20 مقابلة مفصلة مع الأشخاص المشاركين في تفعيل نظام مراقبة التراخوما من جميع مستويات النظام الصحي. حُدث إطار ترميز مواضيعي ثلاثي المستويات من ثلاثة مستويات باستخدام منهج استقرائي في المقام الأول، ولكنه سمح أيضًا بنهج تكراري أكثر، اعتمد على جوانب نظرية متأصلة.

نتائج: أثناء تفعيل خطة المراقبة في غانا ، كان هناك عدد من التعديلات (مقارنة بتوصيات منظمة الصحة العالمية) ، بما في ذلك:

(أ) إدراج مراقبة التراخوما النشطة في نهج المراقبة السلبي ، بالمقارنة مع "بأمراض النمو الداخلي لرموش العين (نمو الرموش داخل الجفن)" وحدها. التحديات التي تعترض تغطية التنفيذ وبنية التقارير غير المحددة التي أعاقت الفعالية ؛

(ب) اختيار عشوائي، وارتفاع في عدد المواقع المختارة لجزء المراقبة الفعال. هذا يفتقر على الأرجح إلى القوة المكانية الزمانية التي تُمَكّن من تعيين عودة الأعراض بعد تَحسّن في الوقت المناسب،

(ج) عمليات بحث مُستهدفة من الباب للباب لأحوال "النمو الداخلي للرموش"، قادها ممرضو عيون. منهجية ناجحة لتعيين حالات "نمو داخلي للرموش" ولكن بكثافة الموارد،

(د) نظام رفيقي بين ممرضي عيون لدعم المهارات التقنية في بيئة تَخلُصيّة حيث أن خسارة المهارات التشخيصية، والجراحية سبب قلق.

لم تأخذ الإستراتيجية في الحسبان فقدان الكفاءة لدى الموظفين ذوي الخبرة.

استنتاجات: طورت غانا نظام مراقبة شاملًا تجاوز توصيات منظمة الصحة العالمية، لكن من المحتمل أن تؤدي المشكلات المتعلقة بالحساسية، والخصوصية إلى الاستخدام غير الفعال للموارد. هناك حاجة إلى تقييم استراتيجيات المراقبة المستهدفة المُحسَّنة لتحديد عمليات البحث عن حالات الإصابة بالنمو الداخلي للرموش. يجب أن تعالج الاستراتيجيات التغيرات السياقية التي تنشأ نتيجة لانخفاض انتقال العدوى ، مثل فقدان المهارات الجراحية.

Translated from English version into Arabic by Sarah Merlin, Revised by Blandine Mathey, through

TRANSLATORS WITHOUT BORDERS

加纳沙眼预检验监测战略的措施调整:一项定性评估

Laura Senyonjo, Agatha Aboe, Robin Bailey, David Agyemang, Benjamin Marfo, Seth Wanye, Elena Schmidt, James Addy, Karl Blanchet

摘要

引言：2009 年，根据 WHO 的建议，加纳开始构建沙眼预检验监测计划。该计划旨在通过主动和被动监测，查明活动性沙眼复发情况，确定并管理倒睫患者。本文概述并回顾分析了加纳在 2011 年至 2016 年间所做的措施调整。该评估可能会为一些国家提供可借鉴的经验，有助于它们的沙眼消除进程。
方法：本研究采用了深入访谈和文献综述的综合方法。在 2016 年 1-4 月间，对来自卫生系统各个级别的参与沙眼监测系统运行的 20 名人员进行了深入访谈。借鉴扎根理论，主要通过归纳方法并允许迭代更新的方式构建了一个三层主题框架。
结果：与 WHO 的建议相比，加纳监测计划在实施过程中进行了一些调整，包括：
(i) 与倒睫监测相比，在沙眼被动监测过程中纳入主动监测方式。但患者鉴定、覆盖实现范围和笼统的报告结构等方面存在的问题妨碍了监测效率；
(ii) 随机选择并增加主动监测点。这可能导致由于场地问题无法及时发现复发病例；
(iii) 由眼科护士带队挨家挨户搜索倒睫患者。这是一种有效的查明倒睫的方法，但资源耗费较多；
(iv) 眼科护士之间建立伙伴制度，在消除进程中提供技术支持，但可能存在诊断和手术技能的缺失。该战略没有考虑到经验丰富人员技术水平的缺失。
结论：加纳建立了一套超出 WHO 建议的全面监测系统，但敏感性和特异性存在问题，可能导致资源的低效使用。需要优化监测战略，以查明复发和进行倒睫患者搜索。战略必须考虑到由于疾病传播能力下降而引起的外在变化，例如手术水平的缺失。

Translated from English version into Chinese by Peng Song, edited by Jin Chen

CHINESE CENTER FOR DISEASE CONTROL AND PREVENTION
NATIONAL INSTITUTE OF PARASITIC DISEASES

Adaptations opérationnelles de la stratégie de surveillance de la prévalidation du trachome utilisée au Ghana: évaluation qualitative des succès et des défis

Laura Senyonjo, Agatha Aboe, Robin Bailey, David Agyemang, Benjamin Marfo, Seth Wanye, Elena Schmidt, James Addy, Karl Blanchet

Résumé

Contexte : En 2009, le Ghana a commencé à élaborer un plan de surveillance de la prévalidation du trachome, basé sur les recommandations alors en cours de l'OMS. Le programme visait à identifier une résurgence du trachome actif, ainsi qu'à identifier et gérer les cas de trichiasis par le biais d'approches de surveillance active et passive. Ce document présente et analyse les adaptations auxquelles a procédé le Ghana entre 2011 et 2016. L'évaluation fournira une opportunité d'apprentissage à un certain nombre de pays à mesure qu'ils progressent vers un statut d'élimination.
Méthodes: Une approche de méthodes mixtes a été adoptée, y compris des entretiens approfondis et une étude documentaire. Entre janvier et avril 2016, 20 entretiens approfondis ont été menés avec des personnes impliquées dans la mise en œuvre du système de surveillance du trachome à tous les niveaux du système de santé. Un système de codification thématique à trois niveaux a été développé en utilisant une approche principalement inductive, mais a également permis une approche plus itérative s'appuyant sur des aspects de la théorie ancrée.

Résultats: Au cours de la mise en œuvre du programme de surveillance du Ghana, un certain nombre d'adaptations ont été apportées (par rapport aux recommandations de l'OMS), notamment :

(i) Inclusion de la surveillance du trachome actif dans l'approche de surveillance passive, par rapport au trichiasis seul. Les problèmes liés à l'identification des cas, les difficultés rencontrées dans l'étendue de la mise en œuvre et une structure hiérarchique non spécifique en ont entravé l'efficacité ;

(ii) Sélection aléatoire et augmentation du nombre de sites sélectionnés pour la composante de surveillance active. Cela manquait probablement du pouvoir spatio-temporel permettant d'identifier la recrudescence dans un délai convenable ;

iii) Recherches ciblées des cas de trichiasis effectuées en porte-à-porte par des infirmières ophtalmologistes. Une méthodologie efficace pour identifier les cas de trichiasis mais nécessitant des ressources considérables ;

(iv) Un système de jumelage entre infirmières ophtalmologistes pour soutenir les compétences techniques dans un contexte d'élimination où la perte de compétences diagnostiques et chirurgicales est préoccupante. La stratégie ne tenait pas compte de la perte de compétences au sein du personnel expérimenté.

Conclusions : Le Ghana a élaboré un système de surveillance complet allant au-delà des recommandations de l'OMS, mais des problèmes de sensibilité et de spécificité ont probablement entraîné une utilisation inefficace des ressources. Des stratégies de surveillance ciblées et améliorées visant à identifier la recrudescence et la recherche de cas de trichiasis doivent être évaluées. Les stratégies doivent prendre en compte les changements contextuels résultant du déclin de la transmission, tels que la perte de compétences chirurgicales.

Translated from English version into French by Sarah Merlin, Revised by Blandine Mathey, through

TRANSLATORS
WITHOUT BORDERS

Действующие изменения в неутвержденной стратегии контроля за течением трахомы в Гане: качественная оценка успехов и проблем

Лора Сеньоджо, Агата Абоэ, Робин Бейли, Дейвид Агьеманг, Бенджамин Марфо, Сет Уанье, Элена Шмидт, Джеймс Эдди, Карл Бланчет

Аннотация

Предпосылки: В 2009 г. в Гане началась разработка плана по контролю за течением трахомы на основе действовавших тогда рекомендаций ВОЗ. Цель данного плана было определить активную стадию трахомы, а также определить и контролировать случаи трихиаза с помощью методов как активного, так и пассивного контроля. В данном документе представлена основная информация и обзор изменений, произошедших в Гане в период с

2011 по 2016 гг. В ходе этой оценки будет предоставлена возможность обучения для ряда стран по мере прогресса к достижению статуса ликвидации.

Методы: Был использован смешанный подход, сочетавший в себе детальные интервью и анализ документов. В период с января по апрель 2016 г. было проведено 20 детальных интервью с лицами, вовлеченными в процесс реализации системы контроля за течением трахомы на всех уровнях системы здравоохранения. Трехуровневая рамочная программа была разработана с применением, в основном, индуктивного подхода; допускалось также применение итеративного подхода с фокусом на аспекты граунд-теории.

Результаты: В ходе реализации плана по контролю в Гане в него было внесено несколько изменений (по сравнению с рекомендациями ВОЗ), в том числе:

(i) включение контроля за течением активной стадии трахомы при применении пассивного контроля по сравнению с контролем за течением только трихиаза. Проблемы с выявлением случаев, трудности с реализацией охвата и неспецифичная структура отчетности препятствовали эффективности;

(ii) Случайный выбор и увеличение количества центров, выбранных в качестве подразделений по активному контролю. Вероятно, их было недостаточно в пространственно-временном отношении для того, чтобы своевременного выявлять обострения;

(iii) Целевой поиск пациентов с трихиазом «от двери до двери» под руководством медсестер со специализацией по офтальмологии. Это эффективная, но ресурсоемкая методология по выявлению случаев трихиаза;

(iv) Наличие "системы дружеской помощи" у медсестер по офтальмологии для обеспечения технических навыков в условиях ликвидации, когда утрата диагностических и хирургических навыков вызывает озабоченность. В этой стратегии не принимается в расчет утрата навыков у части опытного персонала.

Выводы: В Гане была разработана всесторонняя система контроля, превосходящая рекомендации ВОЗ, но проблемы с чувствительностью и специфичностью, возможно, привели к неэффективному использованию ресурсов. Необходимо провести оценку улучшенных целевых стратегий контроля для выявления обострений и поиска пациентов с трихиазом. Стратегии должны учитывать контекстуальные изменения, возникающие в результате снижения заражаемости, такими, как утрата хирургических навыков.

Translated from English version into Russian by Veronika Demeshchyk, Revised by Alexander Somin, through

TRANSLATORS
WITHOUT BORDERS

Adaptaciones operacionales de la estrategia de vigilancia previa a la validación del tracoma, empleada en Ghana: una evaluación cualitativa de logros y desafíos

Laura Senyonjo, Agatha Aboe, Robin Bailey, David Agyemang, Benjamin Marfo, Seth Wanye, Elena Schmidt, James Addy, Karl Blanchet

Resumen

Antecedentes: En 2009, Ghana comenzó a diseñar un plan de vigilancia previa a la validación del tracoma, basado en las recomendaciones actuales de la OMS. El plan tenía como objetivo identificar el resurgimiento activo del tracoma e identificar y gestionar los casos de triquiasis, a través de enfoques de vigilancia activa y pasiva. Este documento describe y examina las adaptaciones realizadas, por Ghana, entre 2011 y 2016. La evaluación proporcionará una oportunidad de aprendizaje, para varios países, a medida que avanzan hasta lograr la eliminación.

Métodos: Se adoptó un enfoque de métodos mixtos, que comprendía entrevistas en profundidad y una revisión de documentos. Entre enero y abril de 2016, se realizaron 20 entrevistas en profundidad, con personas involucradas en la puesta en operación del sistema de vigilancia del tracoma, de todos los niveles del sistema de salud. Se desarrolló un marco de codificación temática de tres niveles utilizando un enfoque principalmente inductivo, que también permitió un enfoque más iterativo, que se basó en aspectos de la teoría fundamentada.

Resultados: Durante la puesta en marcha del plan de vigilancia de Ghana hubo una serie de adaptaciones (en comparación con las recomendaciones de la OMS), entre ellas cabe citar las siguientes:

(i) Inclusión de la vigilancia del tracoma activo, en el enfoque de vigilancia pasiva, en comparación con el caso de la triquiasis. Las cuestiones relacionadas con la identificación de casos, los desafíos relacionados con la implementación de la cobertura y una estructura no específica de presentación de informes dificultaron la eficacia;

(ii) Selección aleatoria y aumento del número de sitios seleccionados, para el componente de vigilancia activa. Esto probablemente carecía del poder espacio-temporal, para poder identificar el recrudecimiento de manera oportuna;

(iii) Búsquedas específicas puerta a puerta de casos de triquiasis, dirigidas por enfermeras oftálmicas. Una metodología eficaz para identificar casos de trichiasis, pero con un uso intensivo de recursos;

iv) un sistema de acompañamiento entre las enfermeras oftalmológicas, para apoyar las aptitudes técnicas en un entorno de eliminación, en el que la pérdida de las aptitudes diagnósticas y quirúrgicas es un motivo de preocupación. La estrategia no tuvo en cuenta la pérdida de competencia, dentro del personal experimentado.

Conclusiones: Ghana desarrolló un sistema de vigilancia integral, que excedió las recomendaciones de la OMS, pero las problemáticas relacionadas a la sensibilidad y especificidad probablemente condujeron a un uso ineficiente de los recursos. Es necesario evaluar las mejores estrategias de vigilancia específicas, para la identificación del recrudecimiento y la búsqueda de casos de triquiasis. Las estrategias deben abordar los cambios contextuales que surgen como resultado de la disminución de la transmisión, como lo es la pérdida de las habilidades quirúrgicas.

Translated from English version into Spanish by María Luz Puerta, Revised by Rosanna Lenci, through

TRANSLATORS
WITHOUT BORDERS
